# Network pharmacology and biological verification of morusin's therapeutic mechanisms in inhibiting nasopharyngeal carcinoma growth

**DOI:** 10.7150/jca.97044

**Published:** 2024-07-16

**Authors:** Zhang Peng, Ran Hong, Yang Dunhui, Wang Zhen, Wu Yongjin, Zeng Xianhai

**Affiliations:** Department of Otolaryngology, Longgang Otolaryngology Hospital & Shenzhen Key Laboratory of Otolaryngology, Shenzhen Institute of Otolaryngology, Shenzhen, Guangdong, China.

**Keywords:** morusin, nasopharyngeal carcinoma, network pharmacology, therapeutic pathways.

## Abstract

Nasopharyngeal carcinoma (NPC) presents a significant therapeutic challenge due to its aggressive nature and limited treatment options. Although morusin, a compound found in traditional Chinese medicines, exhibits significant tumor-inhibiting properties, its specific effects on NPC proliferation remain unclear. This study aims to elucidate the inhibitory effects of morusin on NPC survival and proliferation while exploring the underlying mechanisms through the utilization of network pharmacology, molecular docking, and experimental validation *in vitro* and *in vivo*. Network pharmacology analysis identified 117 potential targets of morusin against NPC, with 8 hub targets including AKT1, BCL2, CASP3, CTNNB1, ESR1, HSP90AA1, MMP9, STAT3, and the IL-17 signaling pathway. Further investigation of public data indicated that the expression levels of BLC2, CASP3, CTNNB1, HSP90AA1, and STAT3 in NPC tissue were significantly elevated compared to normal nasopharyngeal tissue. Docking studies exposed robust binding activity between morusin and key gene molecules. Additionally, biological assays demonstrated that morusin effectively inhibits NPC growth both *in vivo* and *in vitro*. Through a comprehensive investigation, this study identified the pharmacological mechanisms essential for morusin-induced inhibition of NPC growth by targeting multiple molecular targets and signaling pathways. These findings show the potential to contribute to the development of novel clinical agents for treating NPC.

## Introduction

Nasopharyngeal carcinoma (NPC) belongs to the category of head and neck squamous cell carcinoma (HNSCC) 1, displaying distinctive epidemiological characteristics, including regional, racial, and sex-specific patterns 2. According to the World Health Organization, China accounts for 80% of global nasopharyngeal cancer cases. Additionally, a notable aspect of NPC is its pronounced male predominance, with males being two to four times more likely to develop the disease than females in high-risk populations 3. Despite a global decline in NPC incidence in recent years, clinical diagnosis often occurs during the intermediate and advanced stages of the disease due to the subtle nature of early symptoms 4. While NPC exhibits sensitivity to conventional radio/chemotherapy, its 5-year overall survival rate remains suboptimal, ranging from 32% to 62% 5. Therefore, elucidating the molecular mechanisms driving NPC progression is imperative for the development of targeted and efficacious pharmaceutical interventions.

Morusin, a flavonoid derived from the Morus plant, has garnered attention for its exceptional antioxidant capabilities when compared to other flavonoids, owing to its distinct chemical structrue and bioactive properties 6. Previous studies have observed its diverse pharmacological effects, including antibacterial, antioxidant, and anti-inflammatory properties 7. Particularly, morusin has emerged as a modulator of cellular processes implicated in apoptosis, anti-proliferation, and autophagy, exerting its influence through multiple signaling pathways 8. While laboratory studies have shown morusin's potential to inhibit the development of breast, ovarian, and colon cancers primarily by inducing apoptosis 9, the precise mechanisms underlying its anticancer properties remain incompletely understood. Hence, investigating the specific cancer types targeted by morusin and its mechanisms of action is crucial.

In line with this imperative, the present study aims to evaluate morusin's anticancer efficacy against human NPC both *in vitro* and *in vivo*. Moreover, the concept of network pharmacology was first introduced by Prof. Hopkins in 2007^10^, proposing that the pharmacological effects of drugs may involve complex network interactions rather than simple point-to-point interactions 11. By elucidating the connections between disease-related targets and drug targets, network pharmacology offers a promising research approach to drug discovery and development. Thus, leveraging the concept of network pharmacology, our study seeks to explore the potential mechanism by which morusin inhibits NPC proliferation. Additionally, the inhibitory effect of morusin was assessed *in vitro* using NPC cell lines (CNE1 and CNE2), and *in vivo*. The present findings provide a promising approach aimed at advancing the comprehensive application of morusin in both drug development and treatment strategies for NPC, addressing a critical unmet need in the field of oncology.

## Methods and Materials

### Usage of PubChem

The molecular and pharmacological properties data of morusin was obtained from PubChem Database, with the search term 'morusin'.

### Identification of NPC- and morusin-related target genes

Morusin-related targets and NPC-associated targets were collected from several public databases, including The Comparative Toxicogenomics Database (CTD) 12, PharmMapper 13, SwissTarget Prediction 14, Pharmgkb, and GeneCards, Online Mendelian Inheritance in Man (OMIM), Therapeutic Target Database (TTD) 15, DisGeNET (v7.0), respectively.

To identify genetic targets associated with NPC, data from several public databases were collected, including The Comparative Toxicogenomics Database (CTD) 12, PharmMapper 13, SwissTarget Prediction 14, Pharmgkb, and GeneCards. The keyword “nasopharyngeal carcinoma” was used to obtain the gene targets related to the disease. For morusin-related targets, data from Online Mendelian Inheritance in Man (OMIM), Therapeutic Target Database (TTD) 15, and DisGeNET (v7.0) were used. This step provided a comprehensive list of genes used for further investigation that may play a role in the interaction between morusin and NPC. The molecular and pharmacological properties of morusin were obtained from PubChem database (CID: 5281671), which also provided the molecular structure of morusin.

### Protein-protein interaction (PPI) network construction and hub target analysis

Overlapping target genes of NPC and morusin were detected by intersecting the related genes previously identified. These targets were screened and visualized by VennDiagram R package. Subsequently, the PPI network was constructed using the STRING (12.0) database with a medium confidence threshold of 0.4^16^. The PPI network was visualized and analyzed using Cytoscape (3.10.1) 17 with CytoHubba 18 and MCODE plugin 19, enabling the identification of the hub targets within the network. We use a Degree Cutoff of 2, a Node Score Cutoff of 0.2, a K-Core of 2 and a Max.Depth of 100 as a threshold to filter the hub targets. The hub targets were then visualized by VennDiagram R package.

### Pathway enrichment analysis

Candidate hub genes were subjected to Disease Ontology (DO), Gene Ontology (GO) and Kyoto Encyclopedia of Genes and Genomes (KEGG) pathway enrichment analyses using the clusterProfiler package R 20 to obtain the biological functions and pathways associated with NPC and morusin target genes.

### Molecular docking

Molecular docking studies were conducted to investigate the interaction between morusin and the identified hub targets. Briefly, the molecular structures of hub targets were obtained from Protein Data Bank (PDB), including AKT1 (PDB-ID:1h10), BCL2 (PDB-ID:1g5m), CASP3 (PDB-ID:1nme), CTNNB1 (PDB-ID:2z6h), ESR1 (PDB-ID:2bj4), HSP90AA1 (PDB-ID:1byq), MMP9 (PDB-ID:1gkc), and STAT3 (PDB-ID:6njs). Online CB-Dock2 database was used to run the molecular docking simulation between morusin and the hub targets.

### Single-cell RNA-seq dataset collection and processing

Single-cell RNA sequencing (scRNA-seq) data of NPC was downloaded from NCBI Gene Expression Omnibus (GEO) with the accession number GSE152048 and processed using the Tumor Immune Single-cell Hub 2 (TISCH2) database 21 online analysis. Cells were visualized by the uniform manifold approximation and projection (UMAP) method, facilitating the visualization of distinct cell populations and their relationships.

### NPC cell culture

CNE1 was obtained from School of Basic Medical Sciences, Guangzhou University of Chinese Medicine, China. CNE2 was obtained from College of Pharmacy, Guilin Medical University, China. Cells were cultured and maintained in Dulbecco's Modified Eagle Medium (DMEM) supplemented with 9% fetal bovine serum (FBS) and 1% penicillin/streptomycin at 37 °C in a humidified incubator (Thermo Fisher Scientifc) with 5% CO_2_. Reagents were obtained from Gibco (Thermo Fisher Scientific).

### Cell viability assay

To determine cell viability, CNE1 or CNE2 cells were seeded in 96-well plates, after incubation overnight, the cells were treated with the indicated concentrations of different agents for the indicated hours. Following these incubation conditions, cell viability was assessed using a standard colorimetric assay by adding 10 μL Cell Counting Kit-8 reagent (MedChemExpress) to each well. After 2 hours of incubation at 37 °C, the supernatant was discarded and the absorbance was measured at a wavelength of 450 nm using a microplate reader (Molecular Devices). Cell viability was calculated as a percentage relative to untreated control cells. Morusin (CAS No.:62596-29-6) was purchased from MCE Company.

### Colony formation

The colony formation assay was performed to investigate the long-term inhibitory effect of morusin on NPC cell proliferation. CNE1 or CNE2 cells were seeded at a density of 500 cells/well in 6-well plates and incubated overnight. Subsequently, the cells were treated with varying concentrations of morusin (0, 1, 3 μg/mL). Following 2 weeks of incubation, the cultures were fixed with 4% paraformaldehyde and stained with crystal violet to visualize cell growth. Colonies containing more than 50 cells were counted.

### Tumor xenograft experiments

To further assess the inhibitory effects of morusin on NPC cell growth in an *in vivo* setting, tumor xenograft experiments were conducted in nude mice of 5 weeks old. First, mice (n=5) were injected subcutaneously with CNE1 or CNE2 cells. The experimental groups of mice were injected intraperitoneally with morusin (10 mg/kg) every two days. The tumor volume was measured using the formula: volume (mm^3^) = width^2^ × length/2. The animals were euthanized and the tumors were extracted. All experiments were approved by the Animal Experimental Ethics Committee of Shenzhen Institute of Otorhinolaryngology and the ARRIVE guidelines drawn up by the National Centre for the Replacement, Refinement and Reduction of Animals in Research (NC3RS).

### Statistical analysis

Statistical analysis was performed using GraphPad Prism software. Student's t-test was used to compare two groups, and multiple groups were compared using one-way ANOVA. Cell viability and tumor volume were examined by two-way ANOVA. Data were expressed as the mean ± standard error of the mean (SEM). Each experiment was repeated independently at least three times and p-value (p) < 0.05 was considered significant.

## Results

**Figure 1:**
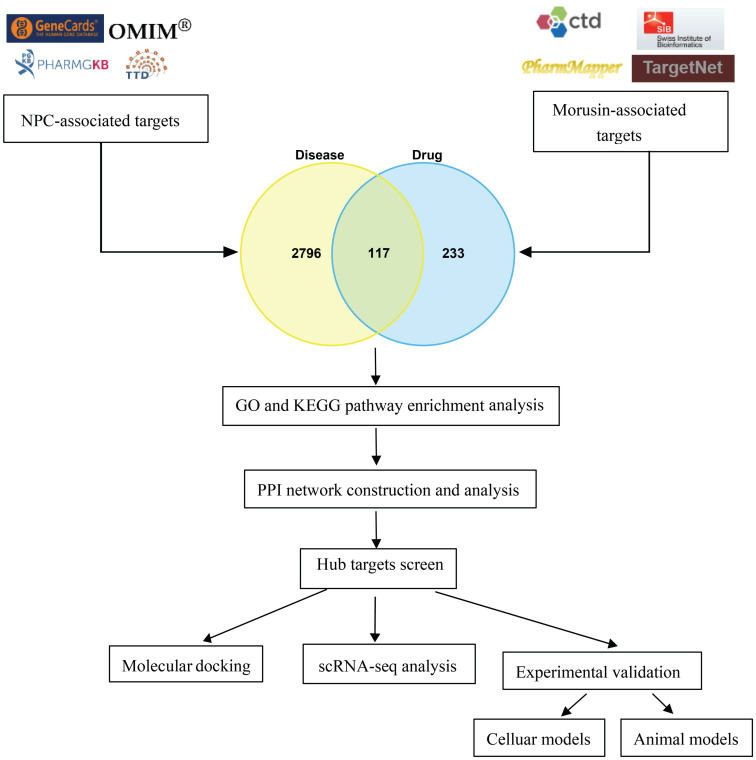
The workflow chart of this study.

As shown in Fig.1, a bioinformatics diagram illustrating the optimized target genes by network pharmacology used to illustrate the anti-growth effect of morusin in NPC cells.

### Identification of NPC-related target genes and signaling pathways

**Figure 2:**
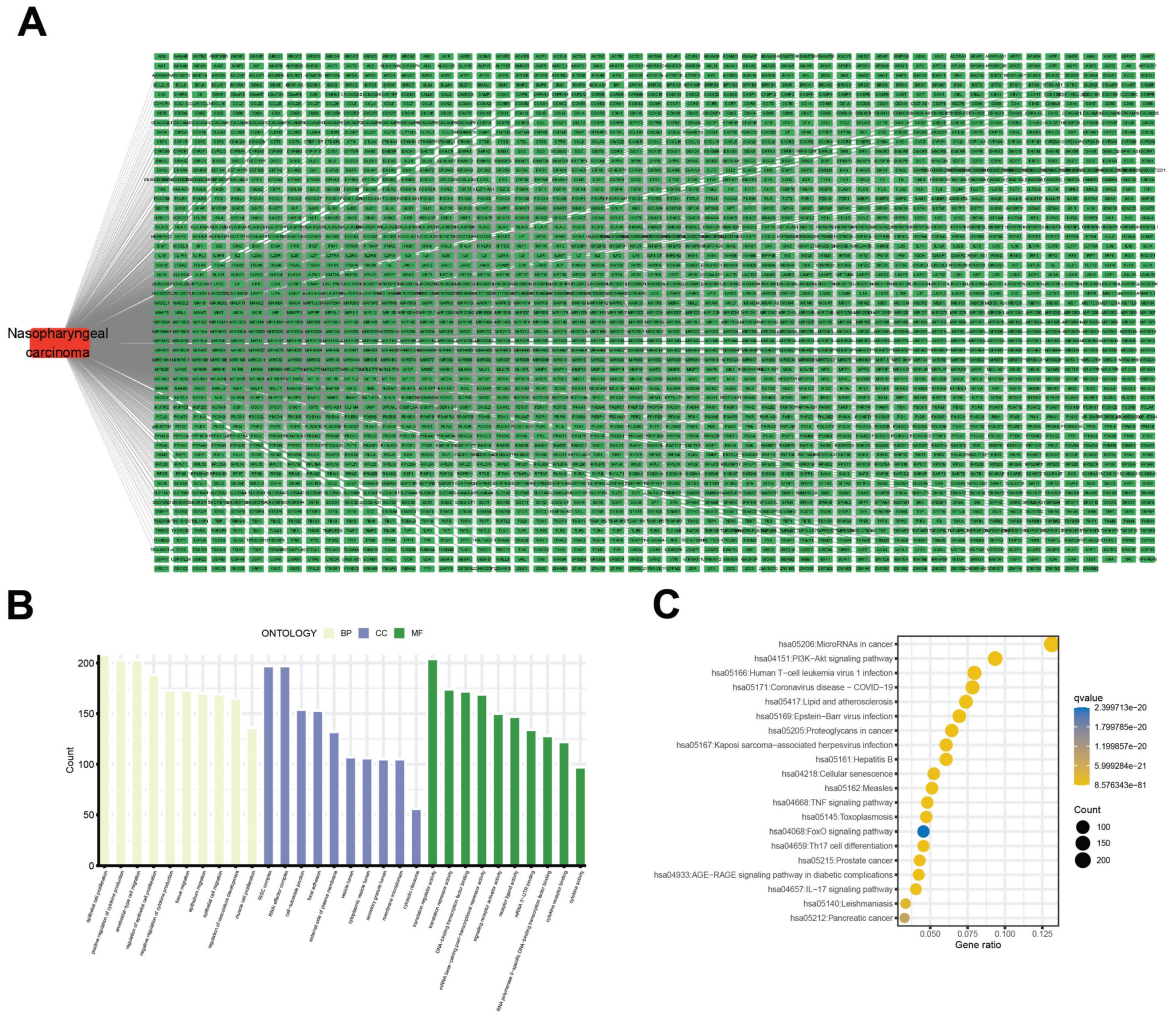
Identification of NPC-related targets and pathways. (A) 2913 NPC-associated targets, the red round rectangle and cyan-green round rectangles represent NPC and NPC-associated targets, respectively. (B) The 10 representative terms with the lowest p-value of biological processes (BPs), cellular components (CCs), and molecular functions (MFs) of the GO enrichment analysis (p < 0.05). (C) The 20 significantly enriched KEGG pathways of NPC-associated targets (p < 0.05).

A bioinformatics analysis of genetic expression datasets was conducted to identify potential novel therapeutic targets for NPC. Initially, a total of 2913 NPC-associated target genes were collected using data from GeneCards, OMIM, TTD, and Parmgkb databases (Fig. 2A). Subsequent analyses using GO and KEGG pathway enrichment revealed 10 significantly enriched items that represent potential therapeutic pathways (Fig. 2B and C). Specifically, these results showed that “epithelial cell proliferation”, “regulation of epithelial cell proliferation”, and “translation regulator activity” were the most significantly enriched items in biological processes, cellular components, and molecular functions, respectively. Additionally, KEGG pathway enrichment analysis showed that “MicroRNAs in cancer” and “Phosphatidylinositol 3-kinase/protein kinase B (PI3K-AKT) signaling pathway” comprised the largest number of targets (Fig. 2C).

### Identification of morusin-related targets and signaling pathway

**Figure 3:**
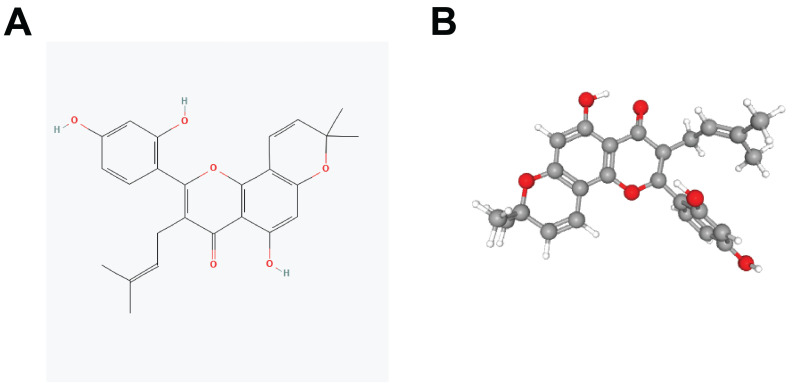
Constitutional formula for morusin.

**Figure 4:**
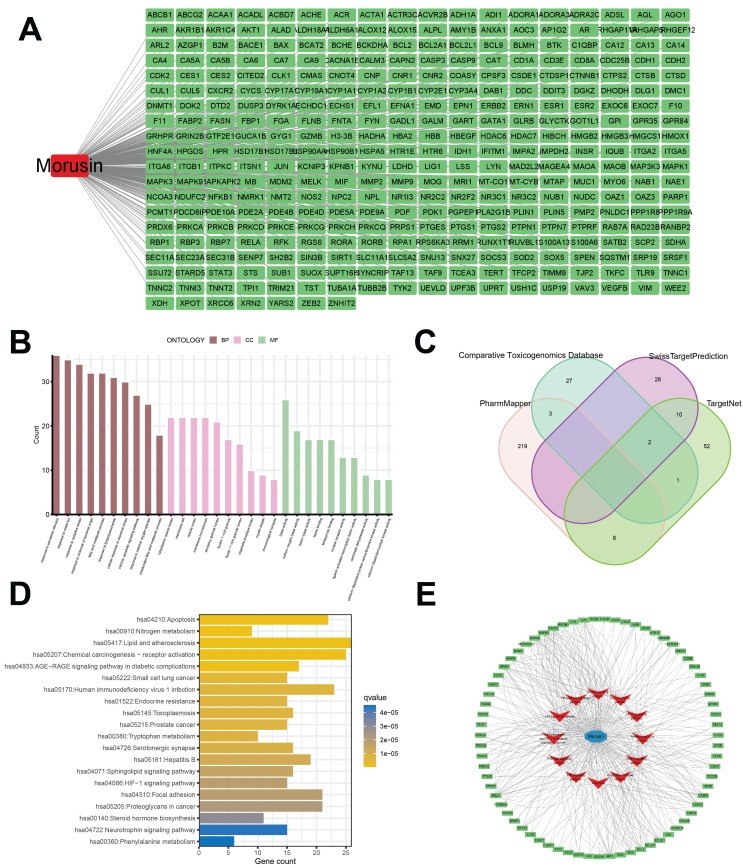
Identification of morusin-related targets and pathways. (A) 350 morusin-associated targets, the red round rectangle and cyan-green round rectangles represent morusin and morusin-associated targets, respectively. (B) Morusin-related targets were visualized by a Venn diagram, incorporating data from PharmMapper, CTD, SwissTargetPrediction, and TargetNet databases. (C) The 10 representative terms with the lowest p-value of biological processes (BPs), cellular components (CCs), and molecular functions (MFs) of the GO enrichment analysis (p < 0.05). (D) The 20 significantly enriched KEGG pathways of morusin-associated targets (p < 0.05). (E) Morusin-target-pathway network with morusin, pathways, and enriched targets represented as a green round rectangle, red V nodes, and blue circles, respectively.

Constitutional formula for morusin was shown in Fig. 3A and B. Next, we identified key genes and signaling pathways that may be affected by morusin. A total of 350 morusin-associated targets were collected using PharmMapper, CTD, SwissTargetPrediction, and TargetNet databases. (Fig. 4A and B). Following GO and KEGG pathway enrichment analyses, results showed that “response to xenobiotic stimulus”, “response to oxidative stress”, and “intrinsic apoptotic signaling pathway” were the most significantly enriched items in biological processes, cellular components, and molecular functions, respectively (Fig. 4C). Moreover, the KEGG pathway enrichment analysis detected the involvement of pathways such as “apoptosis” and “lipid and atherosclerosis” which comprised a higher number of targets (Fig. 4D). The morusin target pathway network is presented in Fig. 4E and depicts the 12 pathways and 178 nodes, including significant pathways like MAPK, IL-17, and NF-κB.

### Identification of the anti-NPC comprehensive pathway analysis of morusin

**Figure 5:**
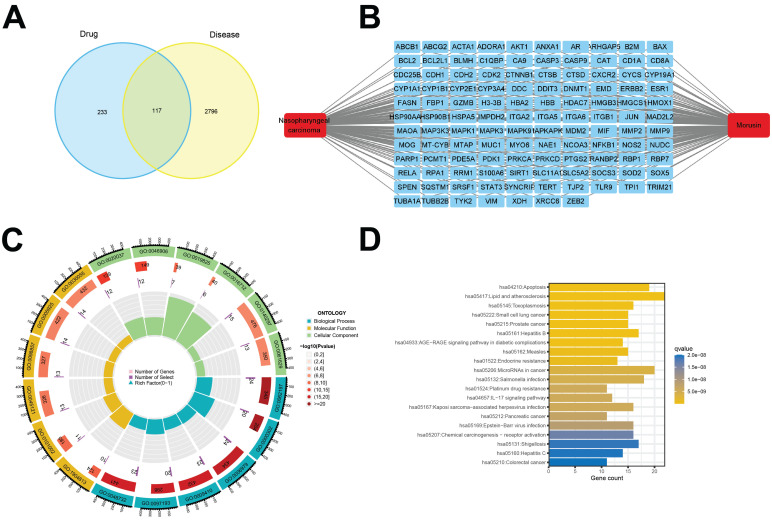
Identification of morusin potential therapeutic pathways. (A) 117 common targets of morusin and NPC-associated targets were visualized by a Venn diagram. (B) 117 common targets of morusin and NPC-associated targets, the red round rectangle and cyan-green round rectangles represent morusin or NPC and morusin or NPC-associated targets, respectively. (C) The 18 significantly enriched GO pathway terms of 117 intersection targets. (D) The 20 significantly enriched KEGG pathway terms of morusin and NPC-associated targets (p < 0.05).

To further identify potential therapeutic pathways by which morusin exerts its effect on NPC cells, a total of 117 intersection targets were screened from the previous analyses (Fig. 5A and B, Table S1). The resulting enriched GO and KEGG pathway terms are shown in Fig. 5C and D. Of note, pathways such as “IL-17 signaling pathway”, “HIF-1 signaling pathway”, and “TNF signaling pathway” emerged as the key enriched pathways (Table S2 and S3), strongly suggesting their involvement in mediating morusin's effects on NPC cells.

**Figure 6:**
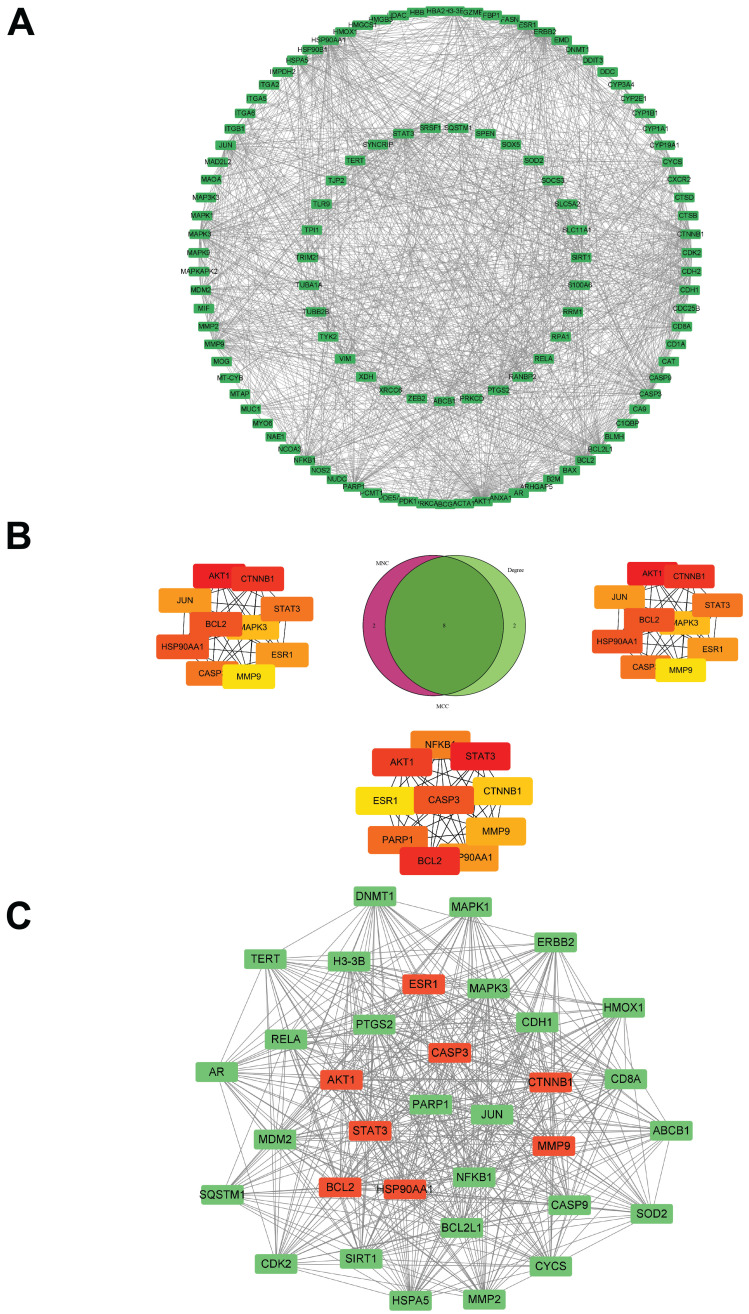
PPI network of morusin with the core targets in NPC setting. (A) The PPI network of morusin in NPC. (B) Ten hub targets were screened by Venn diagram. (C) The most significant module was obtained from MCODE analysis and the red circles represent hub targets.

**Figure 7:**
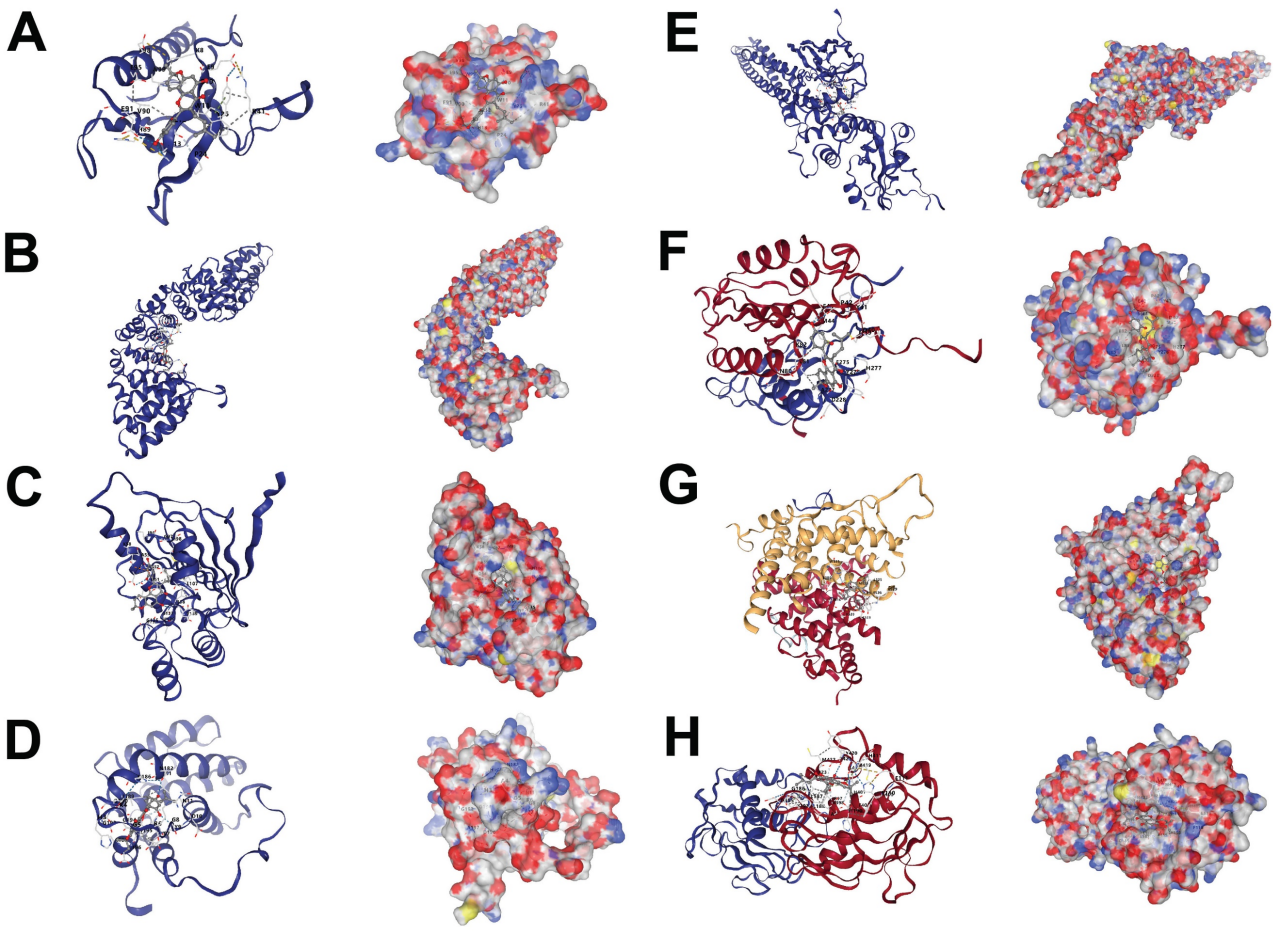
Molecular docking results of morusin and hub targets. (A) Morusin-AKT1. (B) Morusin-CTNNB1. (C) Morusin-HSP90AA1. (D) Morusin-BCL2. (E) Morusin-STAT3. (F) Morusin-CASP3. (G) Morusin-ESR1. (H) Morusin-MMP9.

The core targets of morusin in NPC were investigated through the PPI network (Fig. 6A). By employing algorithms such as MNC, Degree and MCC of CytoHubba, 8 core targets were identified as focal in NPC modulation, including AKT1, CTNNB1, HSP90AA1, BCL2, STAT3, CASP3, ESR1, and MMP9 (Fig. 6B). Interestingly, MCODE analysis discovered a significant module within the network with a score of 29.758 and consisting of 34 nodes and 491 edges. (Fig. 6C), further emphasizing the biological relevance of these targets. Based on the PPI network analysis, eight hub targets were selected to perform molecular docking with morusin. The docking binding energies and binding details of morusin with these hub targets are shown in Fig. 7 and Table 1.

### Distribution of core target expression in NPC tissue

**Figure 8:**
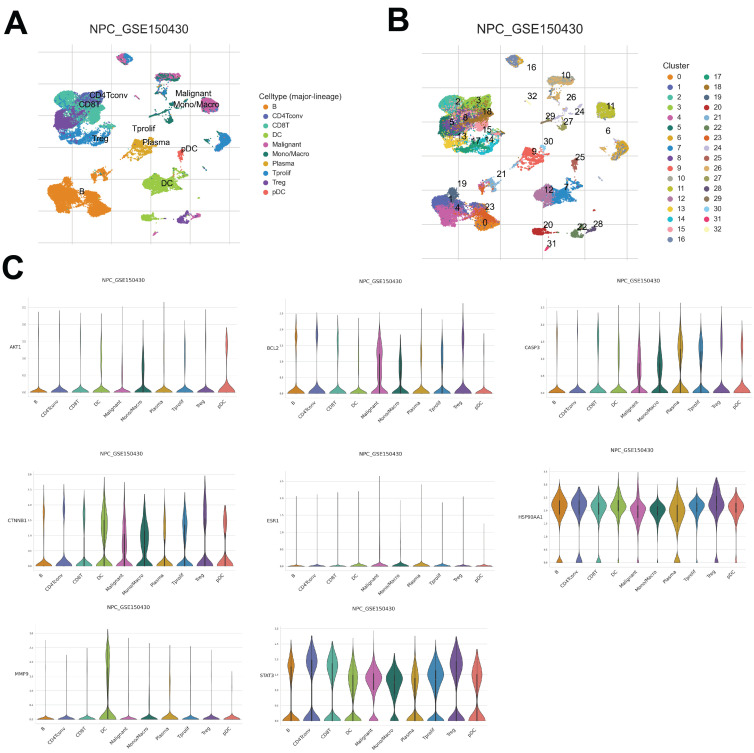
The core targets expression in different NPC cell types. (A, B) UMAP plot showing 10 clusters. (C) The core targets expression in different cell types.

To further understand the distinct expression levels of core targets across different NPC cell types, data from GSE150430 was explored to identify the different cell types of NPC tissues and detect the core target expression. As a result, a total of 32 clusters were acquired and 10 different cell types were classified based on their marker gene expression, including B cells, CD4^+^ conventional T cells, CD8^+^ regulatory T (Treg) cells, dendritic cells (DC), malignant cells, mono/macro cells, plasma cells, T prolif cells, Treg cells, and plasmacytoid DC (PDC) (Fig. 8A and B). Upon examining the gene expression within these cell types, notable trends emerged. For instance, AKT1 exhibited the highest expression levels in PDC of NPC cells (Fig. 8C). CTNNB1, HSP90AA1, CASP3, and BCL2 demonstrated increased upregulation in malignant, mono/macro, T prolif, and Treg cells of NPC, while STAT3 displayed widespread expression in every cell type (Fig. 8C). Moreover, MMP9 exhibited selective expression primarily in DC cells, whereas ESR1 showed relatively lower expression levels across NPC cells (Fig. 8C). Overall, this comprehensive analysis provides insights into the distribution of core target expression within the complex microenvironment of NPC tissue, showing the potential significance of AKT1, CTNNB1, HSP90AA1, CASP3, BCL2, STAT3, and MMP9 in driving NPC pathogenesis, while highlighting ESR1's potential regulatory divergence.

### *In vitro* and *in vivo* validation of morusin effect against NPC

**Figure 9:**
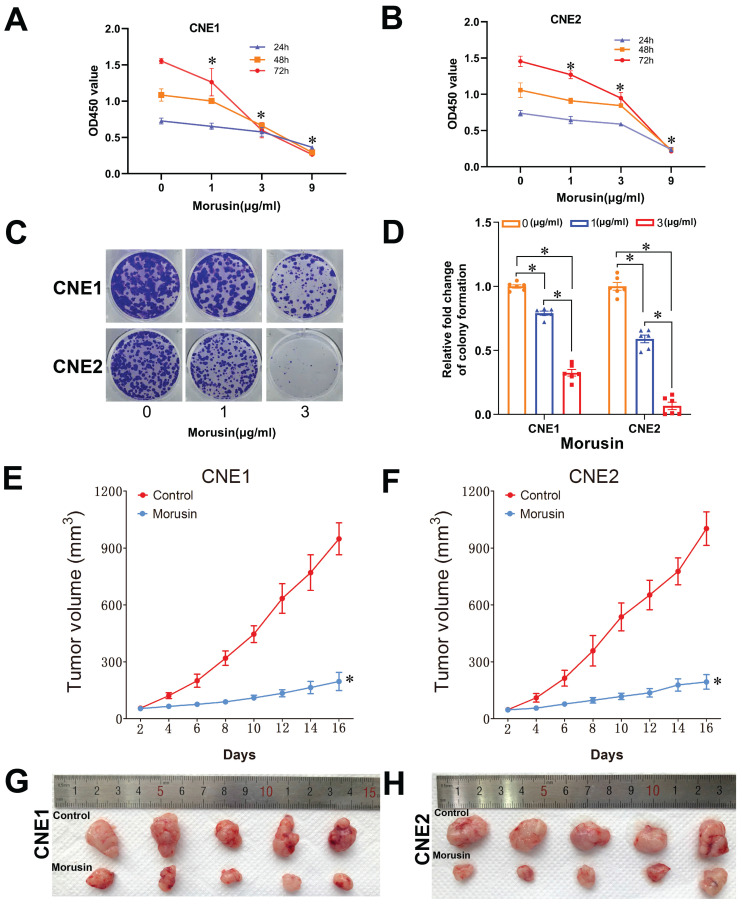
Morusin inhibits cell viability in NPC cells and proliferation in the xenografts mouse model. (A, B) Effect of morusin on the viability of CNE1 and CNE2 cells. (C, D) Representative images and number of colonies formed by CNE1 and CNE2 cells. (E, F) The tumor volume of the xenografts treated with or without morusin (n=5). (G, H) The images of excised tumors in the two groups. * p < 0.05, comparison to the control group.

In order to validate the effects of morusin against NPC as previously predicted by MCODE and CytoHubba analyses, *in vitro* cell proliferation assays were performed using CNE1 and CNE2 cell lines. The results showed that morusin inhibited NPC cell viability in a concentration-dependent manner (Fig. 9A and B). By the colony formation assay, morusin was also found to inhibit cell proliferation ability (Fig. 9C and D).

The results of the network pharmacology and cellular experiments were further fundamented by conducting tumor xenograft experiments of CNE1 or CNE2 cells in nude mice. The mice were afterwards treated with morusin to evaluate the effect of the compound on tumor growth. The results indicated that morusin induced a significant antitumor effect in CNE1 and CNE2 xenograft models, as shown by tumor volume (Fig. 9E and F). The excised tumors are shown in Fig. 9G and H.

## Discussion

The diagnosis of NPC often occurs at advanced stages due to the absence of effective screening protocols and early detection strategies 4. Consequently, many patients present locoregionally advanced or metastatic disease that leads to challenges in treatment and poor outcomes even after comprehensive therapy 22. Conventional salvage therapies, including radiotherapy, chemotherapy, and surgery, frequently yield severe adverse effects and limited efficacy 23. As a result, there is increasing interest in exploring natural products with potential anticancer properties as adjuvant or alternative therapeutic options for NPC 24.

Morusin, a prenylated flavonoid extracted from the root bark of Morus alba, has been extensively studied for its diverse biological properties 25, 26 and previous research has reported its potential antitumor activity against various cancer types, both *in vitro* and animal models studies. These include cervical cancer 27, epithelial ovarian cancer 28, gastric cancer 29, colorectal cancer 30, and hepatocellular carcinoma 7. Given these findings, the objective of this study is to elucidate the potential molecular targets and pathways underlying the therapeutic effects of morusin in the treatment of NPC by employing a combination of network pharmacology, molecular docking, and *in vivo*/*in vitro* experimental validation.

Using publicly available data, the network pharmacology prediction model was employed to forecast drug targets and hub targets through topological analysis. Molecular docking is commonly utilized to evaluate interactions between drugs and targets 31 and was employed to assess the interactions between morusin and the identified targets. Our findings identified 350 morusin-related targets and 2913 targets associated with NPC, suggesting that the “IL-17 signaling pathway” and “PI3K-AKT signaling pathway” may be the primary potential targets implicated in NPC treatment. Additionally, PPI network construction, GO, and KEGG pathway enrichment analyses allowed the identification of eight hub targets from a pool of 117 targets of morusin against the NPC, namely AKT1, CTNNB1, HSP90AA1, BCL2, STAT3, CASP3, ESR1, and MMP.

KEGG pathway analysis suggested that morusin may exert its anti-NPC effects through modulation of the MAPK, IL-17, and NF-κB signaling pathways, with particular involvement of the previously screened key proteins. Of note, AKT is an abundantly expressed oncogene in NPC linked to metastasis and poor prognosis 32. Hence, the present results show that morusin may potentially attenuate AKT's oncogenic activity by interacting with other targets within these pathways. Furthermore, molecular docking studies demonstrated a robust binding affinity between morusin and the hub targets, especially the AKT1 gene. It also adds to proof that AKT1 is a candidate target for the against of NPC with morusin.

Regarding the biological assays, our *in vitro* experiments with CNE1 and CNE2 cell lines confirmed that morusin inhibited NPC cell viability in a dose-dependent manner. This observation aligns with previous studies demonstrating morusin's potential anticancer effect on human hepatocellular carcinoma (HCC) cells, both *in vitro* and *in vivo*, by inducing apoptosis and inhibiting anti-angiogenesis 7. Building on these results, our bioinformatic analysis identifies a potential mechanistic basis for morusin's antitumor and anti-NPC effects, suggesting the induced downregulation of STAT3, AKT1, MMP, and NF-κB 27, 33, 34.

Morusin was reported to reduce STAT3 activity by inhibiting its phosphorylation, nuclear accumulation, and DNA binding activity-induced apoptosis in human prostate cancer cells 33. STAT3 plays a regulatory role in NPC oncogenesis by modulating processes within cancerous cells and their interactions with the tumor microenvironment, which is critical in the initiation, progression, and metastasis of NPC 35. Similarly, the PI3K-AKT pathway is a common oncogenic pathway in a variety of cancers including NPC 8, 36. AKT is a serine/threonine kinase, also referred to as protein kinase B (PKB), that regulates the metabolism of carbohydrates and cell survival, growth, and proliferation 37. Specifically, PI3K-AKT is involved in processes related to tumor initiation/progression and closely related to the clinicopathological features of NPC 38. Notably, phosphorylated AKT overexpression has been identified as a therapeutic target for malignant cancers, as it promotes the proliferation of breast cancer 39. Moreover, matrix metalloproteinase (MMP) expression is regulated by miR-299-3p, leading to significant inhibition of NPC migration and growth 40. In fact, a family of enzymes designated as MMPs has emerged as potential targets for therapy and diagnosis of human tumors, such as breast cancer 41, ovarian cancer 42 and so on. Furthermore, recent reports suggest that Ring finger protein 219 (RNF219) promotes NPC progression through the NF-κB pathway 43. Together, these discoveries emphasize the promising role of morusin as a possible treatment for NPC, providing valuable insights into how it works at the molecular level and pointing towards exciting possibilities for future research and clinical use.

The present study suggests that morusin holds promise as a therapeutic target for the treatment of NPC. Our results indicating that morusin inhibits NPC proliferation through induced apoptosis, which is consistent with previous studies. The significant inhibitory impact observed in NPC cells both *in vitro* and in the mouse model, along with the elucidated action mechanisms of morusin against NPC, provide a new perspective and a robust foundation for further clinical translational research. Moving forward, the underlying mechanisms of morusin against NPC growth will be clarified by high-throughput sequencing, and models of NPC patients' xenografts will be utilized to demonstrate the safety and effectiveness of morusin.

## Conclusion

In conclusion, our comprehensive approach integrating network pharmacology, molecular docking, and experimental validation emphasizes morusin's versatility as a promising candidate for the development of multi-targeted anti-NPC drugs. This research provides a systematic methodology for elucidating the pharmacological mechanisms of morusin in the context of NPC treatment, with implications for future clinical translational investigations.

## Supplementary Material

Supplementary tables.

## Figures and Tables

**Table 1:** Interaction parameters of 8 hub targets and morusin.

Protein	PDB ID	CurPocket ID	Vina score	Cavity volume (Å3)
AKT1	1h10	C3	-6.2	110
BCL2	1g5m	C3	-8.7	285
CASP3	1nme	C3	-7.1	216
CTNNB1	2z6h	C1	-7.7	293
ESR1	2bj4	C4	-8.0	572
HSP90AA1	1byq	C1	-7.9	705
MMP9	1gkc	C2	-9.6	437
STAT3	6njs	C2	-8.0	525
